# Genetic diversity and epidemiology of Genogroup II noroviruses in children with acute sporadic gastroenteritis in Shanghai, China, 2012–2017

**DOI:** 10.1186/s12879-019-4360-1

**Published:** 2019-08-22

**Authors:** Lijuan Lu, Huaqing Zhong, Menghua Xu, Liyun Su, Lingfeng Cao, Ran Jia, Jin Xu

**Affiliations:** Department of Clinical Laboratory, Children’s Hospital of Fudan University, 399 Wanyuan Road, Shanghai, 201102 China

**Keywords:** Norovirus, RdRp/capsid genotypes, Epidemiology, Children, Acute gastroenteritis

## Abstract

**Background:**

Noroviruses (NoVs) are considered an important cause of acute gastroenteritis (AGE) across all age groups, especially in children under 5 years of age. We investigated the epidemiology of noroviruses in outpatient children from the Children’s Hospital of Fudan University in Shanghai, China.

**Methods:**

Stool specimens were collected between January 2012 and December 2017 from 1433 children under 5 years of age with acute gastroenteritis. All samples were analysed by conventional reverse transcription-polymerase chain reaction (RT-PCR) for genogroup II NoVs amplifying both the RNA-dependent RNA polymerase (RdRp) and partial capsid genes. The Norovirus Genotyping Tool v.2.0 (https://www.rivm.nl/mpf/typingtool/norovirus/) was used for genotyping the strains, and phylogenetic analyses were conducted by MEGA 6.0.

**Results:**

From 2012 to 2017, GII NoVs were detected in 15.4% (220/1433) of the samples, with the highest detection rate in children aged 7–12 months (19.2%, 143/746). The seasons with the highest prevalence of GII NoVs infection were autumn and winter. Based on genetic analysis of RdRp, GII.Pe (74.5%%, 137/184) was the most predominant RdRp genotype from 2013 to 2017, while GII.P4 played a dominant role in 2012 (55.6%, 21/36). Among the capsid genotypes, the most prevalent NoV genotype from 2012 to 2017 was GII.4 (74.1%, 163/220). On the basis of genetic analysis of RdRp and capsid sequences, the strains were clustered into − 19 RdRp/capsid genotypes, and 12 of them were discordant, such as GII.Pe/GII.4-Sydney_2012, GII.P12/GII.3, GII.P7/GII.6, GII.Pe/GII.3, and GII.P16/GII.2. Starting with 2013, GII.Pe/GII.4-Sydney_2012 had completely replaced the pandemic GII.P4-2006b/GII.4-2006b subtype and was detected in children across all age groups.

**Conclusions:**

The present study shows high detection rates and the genetic diversity of circulating NoV GII genotypes in paediatric AGE samples from Shanghai. The findings emphasize the importance of continuous molecular surveillance of emerging NoV strains.

## Background

Despite a substantial decrease in the prevalence of gastroenteritis in recent decades, gastroenteritis remains the second most common cause of morbidity and the fourth most common cause of mortality worldwide in children under the age of 5 [1]. After rotaviruses, noroviruses are considered the second most common viral cause of acute gastroenteritis in children [2–5].

*Noroviruses* (NoVs) have a non-segmented positive-strand RNA genome of approximately 7.6 kb that contains three ORFs [6]. ORF1 encodes a polyprotein that is cleaved by the virus-encoded protease into six nonstructural proteins, including the norovirus protease and RNA-dependent RNA polymerase (RdRp). ORF2 encodes the major structural protein (VP1), and ORF3 encodes a minor structural protein (VP2) [7]. NoVs are highly diverse and divided into seven genogroups (GI-GVII), of which GI, GII and GIV have been found in humans, and GII is the most prevalent genogroup in children with acute diarrhoea. GII NoVs can be subdivided into 29 genotypes or 23 genotypes based on the genetic diversity of ORF1 or ORF2, respectively [8, 9]. An increasing number of ORF1/ORF2 combination genotypes of NoVs have been appearing in many areas [10–17]. Taking into account the phylogenetic relationships of both partial ORF1 and ORF2 sequences, a dual nomenclature system for NoVs has been proposed. This dual typing approach can correctly identify genetically different NoV genotypes. In addition, combinant genotypes are better for understanding the molecular epidemiology of NoVs.

Previous studies in Shanghai mainly adopted either ORF1 or ORF2 nomenclature to understand the epidemiology of NoVs in children under 5 years of age [18–22]. Here, we adopted a dual nomenclature system based on both ORF1 and ORF2 to investigate the diversity of NoV GII genotypes in children suffering from acute gastroenteritis who visited the Children’s Hospital of Fudan University in Shanghai from January 2012 to December 2017. Furthermore, we assessed the overall frequency of GII NoVs, seasonal distribution of GII NoVs, and NoV GII genotype distribution by age group.

## Methods

### Study design

Faecal specimens were collected from 1433 children up to 5 years old who visited the outpatient department of the Children’s Hospital of Fudan University and were diagnosed with acute gastroenteritis between January 2012 and December 2017 in Shanghai. Acute gastroenteritis is defined as ≥3 instances of loose stool or looser-than-normal stool within a 24-h period combined with significant changes in the faecal exterior, including a watery or thin paste texture and the presence of mucous; this definition excluded the presence of pus or blood regardless of the presence of fever [20]. Demographic information and clinical diagnoses were collected from the patients’ medical histories. The study was approved by the Institutional Review Board of the Children’s Hospital of Fudan University.

Stool suspensions were prepared as 10% (w/v) in saline solution. Nucleic acid was extracted from 200 μL clarified stool suspensions using a TIANamp Virus DNA/RNA Kit (TIANGEN Biotech (Beijing) Co., Ltd.) according to the manufacturer’s recommendations. The extracted genetic material was submitted to reverse transcription (RT) with a random primer using PrimeScript™ II Reverse Transcriptase (Takara, Biotechnology (Dalian) Co., Ltd.).

cDNA was amplified by PCR for GII NoV genotyping. PCR and sequencing of NoVs were performed using primers targeting the RdRp region of ORF1 (313 bp) and partial capsid region of ORF2 (344 bp) (Table 1). PCR was performed under the following conditions: initial denaturation at 94 °C for 2 min, followed by 35 cycles of 94 °C for 30 s, 55 °C for 30 s, 72 °C for 30 s, and 72 °C for 7 min for a final extension. All amplified cDNA products were electrophoresed on 2.0% agarose gels containing 4S GelRed and visualized by the gel doc EZ imaging system (bio-rad laboratories (Shanghai) co., ltd.). The amplification products of NoV-positive samples were subjected to nucleotide sequencing by Sangon biotech (Shanghai) co., ltd.

**Table 1:** Primers used for Noroviruses genotyping in this study

Primers	Nucleotide position	Polarity	Target	Sequence (5′- 3′)	Size
G2SKF	5058	F	Capsid	CNTGGGAGGGCGATCGCAA	344 bp
G2SKR	5401	R	Capsid	CCRCCNGCATRHCCRTTRTACAT	
289H	4865	R	RdRp	TGACGATTTCATCATCACCATA	331 bp
289I	4865	R	RdRp	TGACGATTTCATCATCCCCGTA	
290H	4590	F	RdRp	GATTACTCCAGGTGGGACTCCAC	
290I	4590	F	RdRp	GATTACTCCAGGTGGGACTCAAC	
290 J	4590	F	RdRp	GATTACTCCAGGTGGGATTCAAC	
290 K	4590	F	RdRp	GATTACTCCAGGTGGGATTCCAC	

Sequences generated from the PCR products of each strain were analysed by using the Norovirus Genotyping Tool v.2.0 (https://www.rivm.nl/mpf/typingtool/norovirus/), where each sequence was assigned a NoV genotype. Phylogenetic analysis of the sequences in our study and of sequences from GenBank was also performed using the maximum likelihood method (Kimura two-parameter model, 1000 bootstrap replications for branch support) in MEGA 6.0.

## Statistical analysis

The difference between NoV detection rates in boys and girls was compared using a two-sided chi-square test in SPSS Statistics v.19.0 (IBM Corp., Armonk, NY, USA), and a *P-*value less than 0.05 was considered statistically significant.

## Results

### Detection and epidemiology of GII NoVs in children

A total of 1433 stool samples were collected from 2012 to 2017 from outpatient children with acute gastroenteritis, of which 897 (62.6%) were boys and 536 (37.4%) were girls. Among these children, 15.4% (220/1433) were infected with the NoV GII genogroup, and the annual detection rates were 25.0% (36/144), 15.3% (22/144), 11.8% (17/144), 15.5% (41/265), 18.8% (59/313) and 10.6% (45/423) from 2012 to 2017, respectively. No significant difference (*P* > 0.05) in the NoV detection rate was observed between boys (16.4%, 147/897) and girls (13.6%, 73/536).

**Fig. 1:**
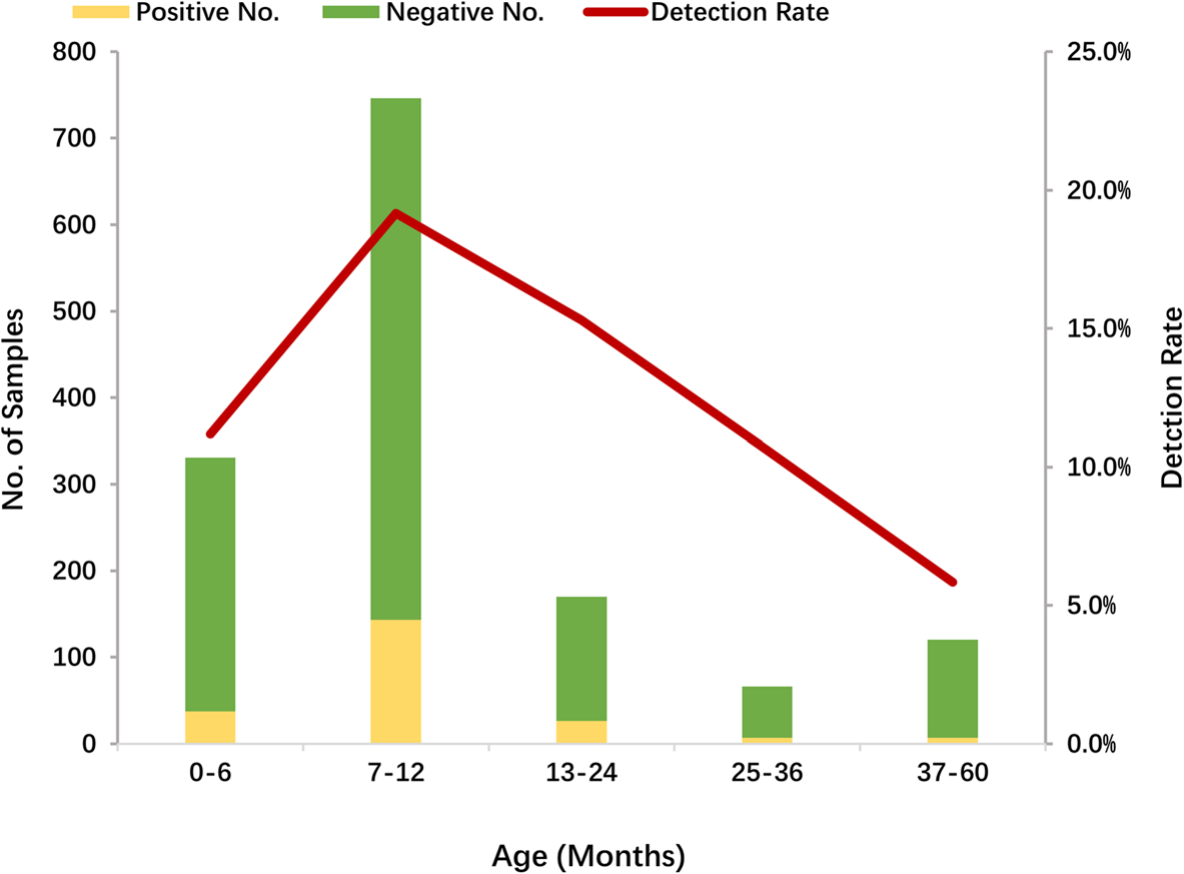
Age distribution of norovirus infection in children under 5 years of age with acute gastroenteritis in Shanghai, 2012–2017

**Fig. 2:**
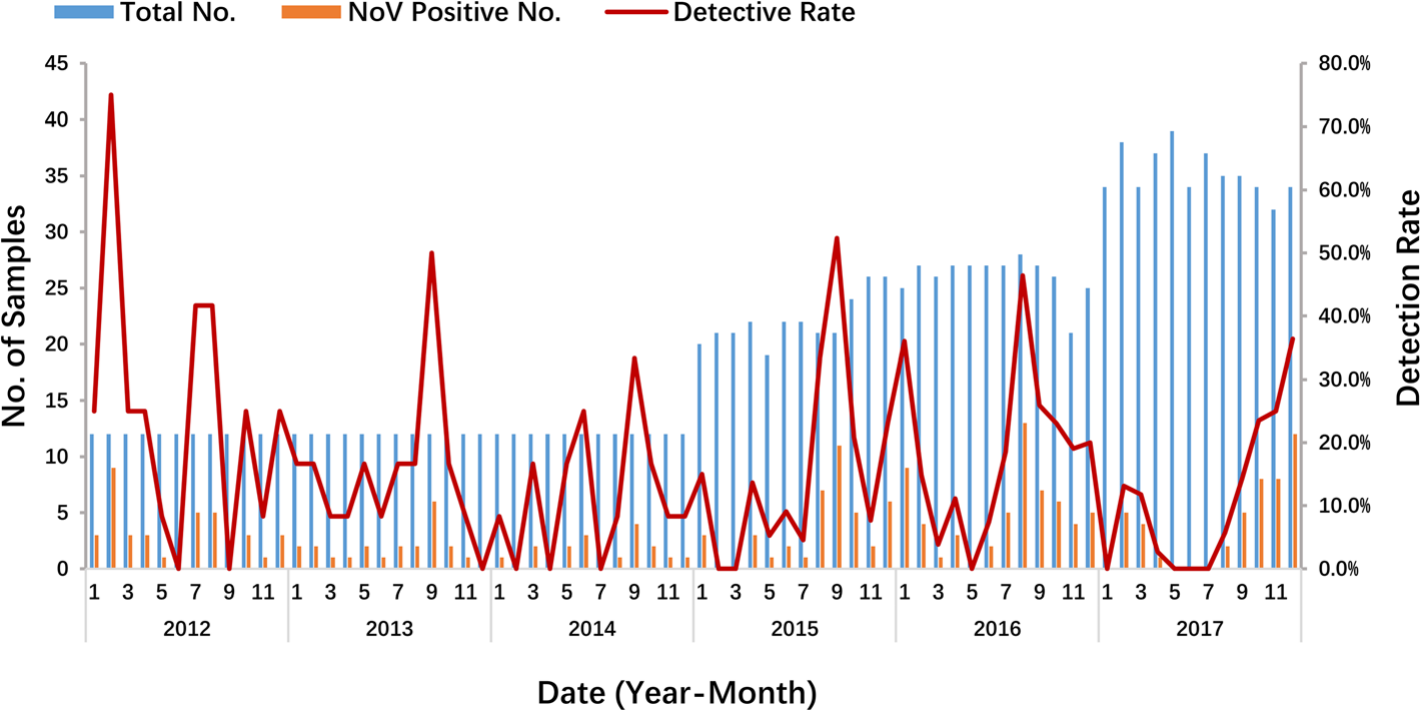
Seasonal distribution of norovirus infection in children under 5 years old with acute gastroenteritis in Shanghai, 2012–2017

The overall prevalence of GII NoV infection in the different age groups ranged from 5.8 to 19.2%. Children in the age group 7–12 months had the highest prevalence (19.2%, 143/746), followed by children aged 13 to 24 months (15.3%, 26/170) (Fig. 1). Most of the children infected with NoVs (93.6%, 206/220) were aged less than 2 years. GII NoV infection was prevalent in all seasons from 2012 to 2017, and the seasons with the highest prevalence of NoV infection were autumn and winter, except for a small peak in the summer of 2012 (Fig. 2).

### NoV GII genotype distribution based on the RdRp region

**Fig. 3:**
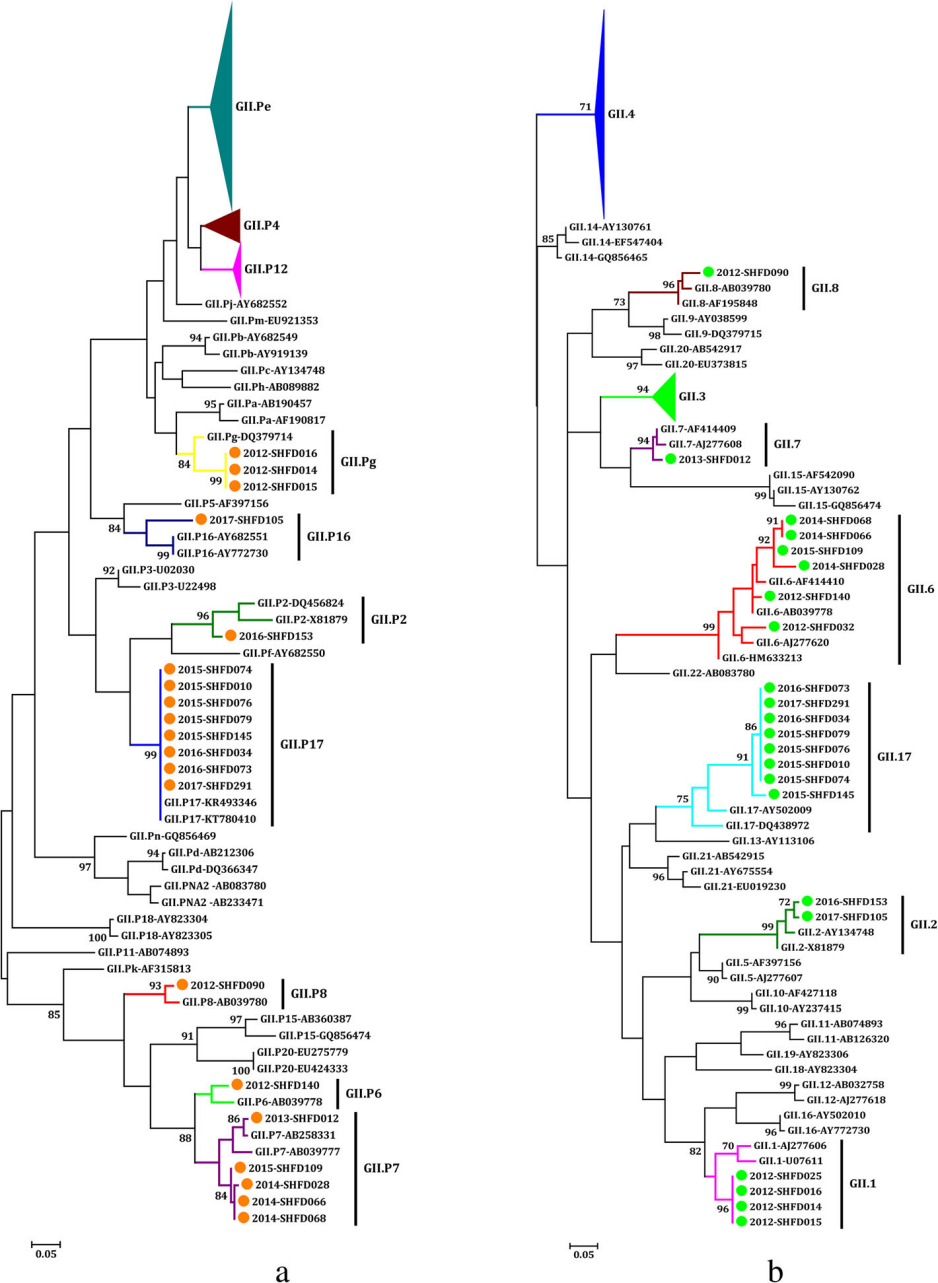
Phylogenetic analysis of GII norovirus based on the partial nucleotide sequences of the viral polymerase (**a**) and capsid regions (**b**). The NoV strains detected in this study are indicated by coloured dots. References NoV genotypes are labelled according to GenBank with their respective accession numbers. The trees were constructed in MEGA 6.0 through the maximum likelihood method using the Kimura 2-parameter model. The bootstrap values (1000 replicates) are indicated in the phylogenetic tree, and values less than 70% are not represented

**Fig. 4:**
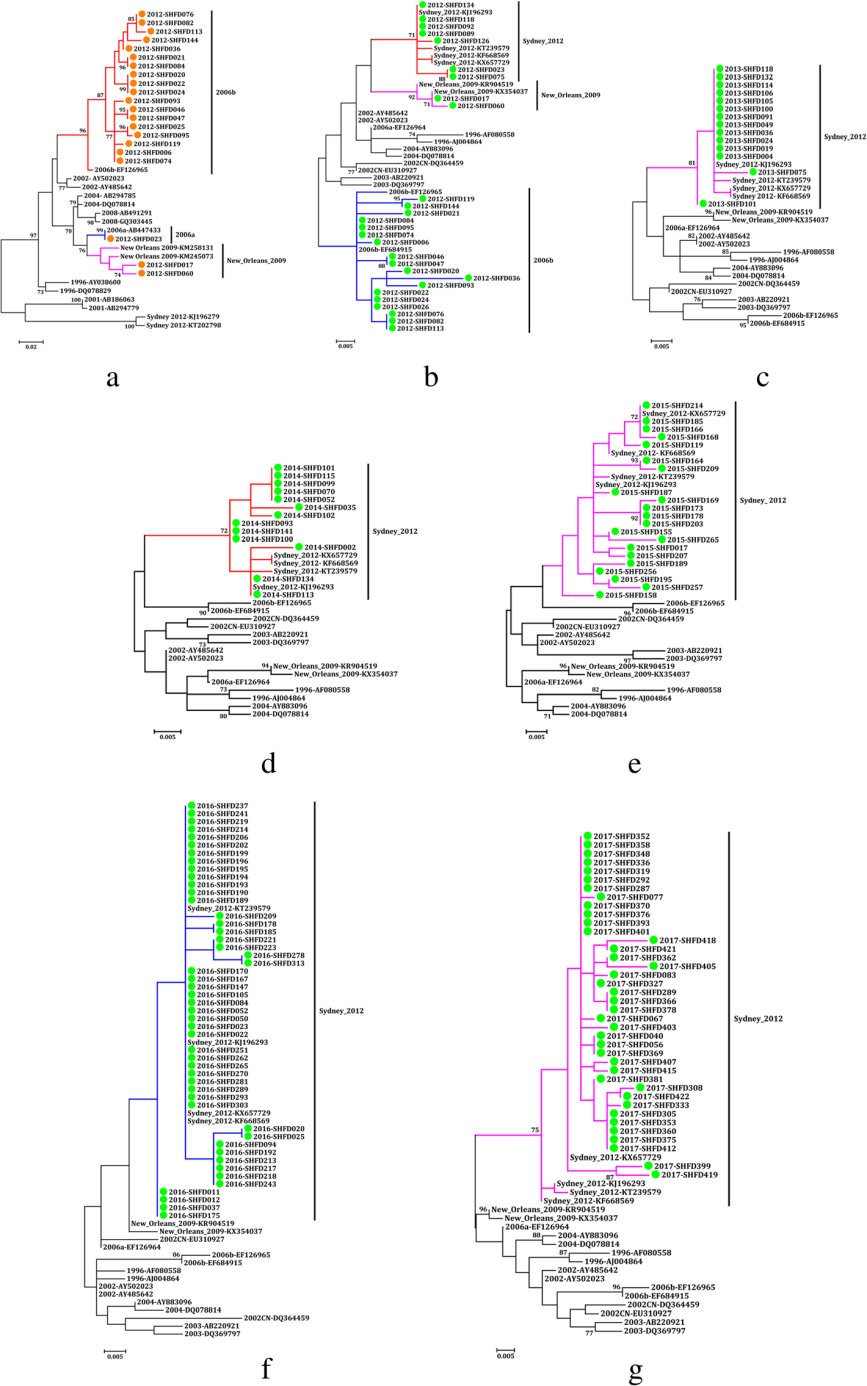
Phylogenetic analysis of GII.4 variants based on the partial nucleotide sequences of the viral polymerase (**a**: 2012) and capsid regions (**b-g**: 2012–2017). References NoV genotypes are labelled according to GenBank with their respective accession numbers. The trees were constructed in MEGA 6.0 through the maximum likelihood method using the Kimura 2-parameter model. The bootstrap values (1000 replicates) are indicated in the phylogenetic tree, and values less than 70% are not represented

When the polymerase region was analysed, the large genetic diversity of the circulating NoV GII strains was observed in the 220 NoV-positive samples. GII.Pe (74.5%, 137/184) was the predominant RdRp genotype from 2013 to 2017, while GII.P4 played a dominant role in 2012 (55.6%, 21/36). The second most prevalent genotype varied from year to year. GII.Pe and GII.P7 were the second most prevalent genotypes in 2012 and 2014, while GII.P12 was the second most prevalent genotype in the rest of the years. GII.P17 was sequenced successfully from eight samples from 2015 to 2017. In addition, many other RdRp genotypes were detected, such as GII.Pg (1.4%, 3/220), GII.P16 (0.5%, 1/220) and GII.P8 (0.5%, 1/220). Three GII.P4 subtypes were detected in only 2012, including GII.P4-2006b (50.0%, 18/36), GII.P4-2006a (2.8%, 1/36) and GII.P4-New_Orleans_2009 (5.6%, 2/36) (Figs. 3 and 4a, Table 2).

**Table 2:** NoV GII genotypes distribution according to the RdRp region of ORF1 or partial region of ORF2 in Children with acute gastroenteritis under 5 years in Shanghai, 2012–2017

Genotypes	2012	2013	2014	2015	2016	2017	Total
	n(m%)						
RdRp region of ORF1							
GII.P2	–	–	–	–	1(1.7)		1(0.5)
GII.P4-2006a	1(2.8)	–	–	–	–	–	1(0.5)
GII.P4-2006b	18(50.0)	–	–	–	–	–	18(8.1)
GII.P4-New_Orleans_2009	2(5.6)	–	–	–	–	–	2(0.9)
GII.P6	1(2.8)	–	–	–	–	–	1(0.5)
GII.P7	–	1(4.5)	3(17.6)	1(2.4)			5(2.2)
GII.P8	1(2.8)	–	–	–	–	–	1(0.5)
GII.P12	2(5.5)	6(27.3)	1(5.9)	14(34.2)	7(11.9)	4(8.9)	34(15.4)
GII.P16	–	–	–	–	–	1(2.2)	1(0.5)
GII.P17	–	–	–	5(12.2)	2(3.4)	1(2.2)	8(3.6)
GII.Pe	8(22.2)	15(68.2)	13(76.5)	21(51.2)	49(83.0)	39(86.7)	145(65.9)
GII.Pg	3(8.3)	–	–	–	–	–	3(1.4)
Total	36(100)	22(100)	17(100)	41(100)	59(100)	45(100)	220(100)
Partial region of ORF2							
GII.1	4(11.1)	–	–	–	–	–	4(1.8)
GII.2	–	–	–	–	1(1.7)	1(2.2)	2(0.9)
GII.3	2(5.6)	7(31.8)	1(5.9)	14(34.2)	7(11.9)	4(8.9)	35(15.9)
GII.4-2006b	18(50.0)	–	–	–	–	–	18(8.2)
GII.4-Sydney_2012	7(19.4)	14(63.6)	13(76.5)	21(51.2)	49(83.0)	39(86.7)	143(65.0)
GII.4-New_Orleans_2009	2(5.6)	–	–	–	–	–	2(0.9)
GII.6	2(5.6)	–	3(17.6)	1(2.4)	–	–	6(2.7)
GII.7	–	1(4.6)	–	–	–	–	1(0.5)
GII.8	1(2.7)	–	–	–	–	–	1(0.5)
GII.17	–	–	–	5(12.2)	2(3.4)	1(2.2)	8(3.6)

n: NoV positive numbers

m: Constituent ratio of each genotype

### NoV GII genotype analysis based on the partial capsid region

On the basis of the partial capsid region, the majority of the NoV GII strains were classified as GII.4 (73.6%, 162/220) from 2012 to 2017, followed by GII.3 (15.9%, 35/220). The other non-GII.4 genotypes included GII.17 (3.6%, 8/220), GII.6 (2.7%, 6/220), GII.1 (1.8%, 4/220), GII.2 (0.9%, 2/220), GII.7 (0.5%, 1/220) and GII.8 (0.5%, 1/220). GII.4 was divided into three subtypes by the Norovirus Genotyping Tool v.2.0. GII.4-Sydeny_2012 (73.9%, 136/184) was the most frequent GII.4 variant from 2013 to 2017, while GII.4-2006b (50.0%, 18/36) was the main variant in 2012. Two sequences detected in samples from 2012 belonged to GII.4-New_Orleans_2009 (Figs. 3 and 4b-g, Table 2).

### Combination genotype of GII NoVs with both RdRp/capsid fragment genes

**Fig. 5:**
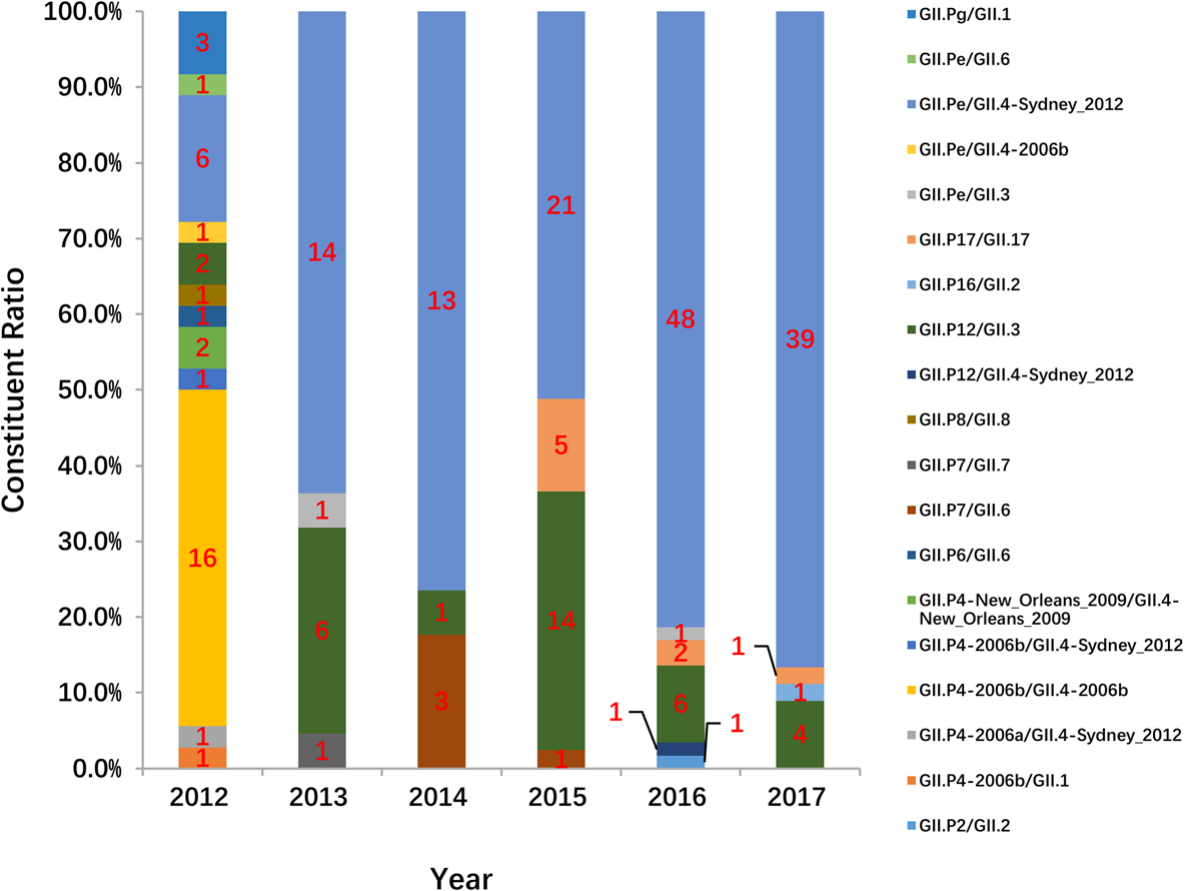
Distribution of NoV RdRp/capsid combination genotypes in children under 5 years old with acute gastroenteritis in Shanghai, 2012–2017

Overall, 19 kinds of RdRp/capsid genotypes were presented according to the dual nomenclature system of NoVs, and discordant RdRp and capsid genotypes were identified in 12 of them, including GII.Pe/GII.4-Sydney_2012, GII.P12/GII.3, GII.P7/GII.6 and GII.P16/GII.2. GII.Pe/GII.4-Sydney_2012 (73.4%, 135/184) was the dominant RdRp/capsid genotype in each year from 2013 to 2017, while GII.P4-2006b/GII.4-2006b (44.4%, 16/36) was the most frequent genotype in 2012. GII.P12/GII.3 was the second most frequent combination genotype in 2013 (27.3%, 6/22), 2015 (34.1%, 14/41), 2016 (10.2%, 6/59) and 2017 (8.9%, 4/45), while GII.Pe/GII.4-Sydney_2012 (16.7%, 6/36) and GII.P7/GII.6 (17.6%, 3/17) were the second most frequent genotypes in 2012 and 2014. Most of the uncommon combination genotypes were detected in 2012, for example, GII.Pe/GII.6, GII.Pg/GII.1 and GII.Pe/GII.4-2006b. In addition, GII.P17/GII.17 was only detected in eight children from 2015 to 2017 (Fig. 5).

### Distribution of NoV GII RdRp/capsid genotype in children of different ages

**Fig. 6:**
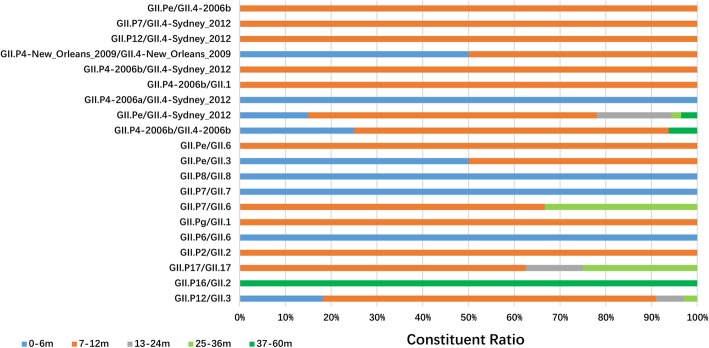
Distribution of NoV RdRp/capsid combination genotypes in children of different ages with acute gastroenteritis in Shanghai, 2012–2017

In this study, only the GII.Pe/GII.4-Sydney_2012 genotype was distributed across all age groups in children. Almost all the NoV RdRp/capsid genotypes were detected in children aged from 7 to 12 months (63.6%, 143/220). Among these genotypes, GII.P2/GII.2, GII.Pg/GII.1, GII.Pe/GII.6, GII.P4-2006b/GII.1, GII.P4-2006b/GII.4-Sydney_2012, GII.P12/GII.4-Sydney_2012, GII.P7/GII.4-Sydney_2012 and GII.Pe/GII.4-2006b were only detected in 7- to 12-month-old children. GII.P4-2006a/GII.4-Sydney_2012, GII.P6/GII.6, GII.P7/GII.7 and GII.P8/GII.8 were only distributed in children aged from 0 to 6 months, and GII.P16/GII.2 was only detected in a child aged 60 months (Fig. 6).

## Discussion

This was a long-term monitoring study of the epidemiology and molecular characteristics of NoVs in children under 5 years of age with acute sporadic gastroenteritis in Shanghai. Herein, the total detection rate of GII norovirus-positive cases (15.4%) among outpatients was lower than that in our previous monitoring data of NoV infections in both outpatients and inpatients [20, 22]. Although the percentage of NoV infections in 2012 (25.0%) was similar to our previous data from 2006 to 2011, the annual detection rate of NoVs was characterized by a fluctuating reduction from 2012 to 2017 [22]. This finding may be associated with the increased publicity on how to prevent NoV infection among people because of the increasing number of outbreaks of NoV infection in many areas in recent years.

From 2012 to 2017, children with GII NoV infection were mainly aged less than 2 years, which is consistent with our previous study and other studies [20, 22–25]. No significant difference in NoV detection rates was found in girls and boys. This finding may imply that gender is not a predisposing factor for NoV infection in children less than 5 years old. In accordance with previous studies that concluded that noroviruses mainly peaked in cold seasons [20, 22, 26–28], our study demonstrated that most NoV infections were detected in autumn and winter. However, in some other areas, NoV-associated diarrhoea had a summer peak or no apparent seasonal peak, which may be connected with an increase in contaminated water and food or other unknown reasons [21, 29].

A great diversity in NoV GII genotypes was identified on the basis of the RdRp region. Among the NoV-positive cases, GII.P4 was the predominant NoV genotype in 2012, which is consistent with our previous data on outpatient children from 2010 to 2011 and western China from 2010 to 2013 [17, 20]. It is interesting to note that the prevalence of GII.P4 abruptly disappeared after 2012, while GII.Pe became the obviously dominant genotype circulating in children under 5 years of age with acute sporadic gastroenteritis from 2013 to 2017. This unexpected increase in GII.Pe from 2013 was also found in Huzhou, China [30]. Based on these results, we speculated that GII.Pe, first detected in the norovirus outbreak of 2008 in Victoria, Australia, has obviously replaced GII.P4 as the leading RdRp genotype in children with sporadic gastroenteritis in Shanghai since 2013 [31]. However, no GII.Pe was detected in Suzhou (China) and western China in 2013 [17, 32]. Close attention should be paid to the spread of this genotype in China in the future. It was surprising that GII.P12 became the second main RdRp genotype instead of GII.Pb from 2012 to 2017. No GII.Pb was detected in this study, although it was the second most predominant RdRp genotype from 2010 to 2011 in Shanghai [20].

Since 2014, GII.17 has become increasingly popular in several major cities in mainland China and other areas in Asia [33–36]. However, only eight GII.P17/GII.17 strains were detected from 2015 to 2017 in our study. The same detection rate of GII.P17/GII.17 in children was also reported in Huzhou and Shanghai [30, 36]. However, adults were more susceptible to GII.P17/GII.17 than children [30, 36]. The reason for this different susceptibility to infection between diverse age groups is still unclear, and further studies need to be conducted to explore the mechanism of this difference.

Similar to the NoV RdRp genotypes, diverse NoV GII genotypes were detected on the basis of the partial capsid gene. Similar to data from Korea (2013–2015), Japan (2008–2014), Chongqing and Suzhou in China (2010–2013), Lusaka Province in Zambia (2012–2013) and Vietnam (2012–2015), GII.4 was the predominant capsid genotype spreading among children from 2012 to 2017 [17, 32, 37–40]. The differences in the predominant genotypes determined by the two sequence-based typing methods indicated the importance of genotyping NoVs simultaneously by both capsid and RdRp genes, which could assist us in comprehensively understanding the epidemiology and evolution of NoVs. Although GII.4-2006b was still the predominant GII.4 variant, GII.4-Sydney_2012 became the main and only GII.4 subtype from 2013 to 2017. GII.4-Sydney_2012 was first reported in Australia, and it has been the most prevalent variant among children worldwide since 2012 [41]. As reported in Bangladesh (2010–2014), Chongqing (2010–2013) and Jiangsu (2010–2013) in China, in Japan (2008–2012) and in Vietnam (2012–2015), GII.3 was the second most prevalent capsid genotype in our study [17, 32, 38, 40, 42].

The analysis of combined NoV GII genotypes conducted in this study demonstrated that 19 different RdRp/capsid genotypes were determined and that 12 of them were distinct in RdRp genotypes and capsid genotypes. All of the discordant RdRp/capsid genotypes were suspected to be recombinant strains, and most of them have been reported elsewhere [14, 16, 17, 23, 37, 41]. Although more analysis of the junction of ORF1 and ORF2 is needed to confirm the recombination site in our study, the results clearly suggest that this phenomenon is very common in NoVs, as observed elsewhere. Furthermore, we observed a change in the circulation pattern of the RdRp/capsid-genotyped strains, with GII.P4-2006b/GII.4-2006b being predominant in 2012, followed by an emergence and predominance of GII.Pe/GII.4-Sydney_2012 starting in 2013. GII.Pe/GII.4-Sydney_2012, first reported in Australia in 2012, has been widespread in South Africa (2012–2013), Iran (2015–2016), Botswana (2013–2015), Korea (2013) and some cities in China (2012–2015) since then [23, 31, 37, 41, 43–46]. It was surprising that, starting with 2013, GII.Pe/GII.4-Sydney_2012 had completely replaced the pandemic GII.P4-2006b/GII.4-2006b subtype, and close attention should be paid to the prevalence of this genotype.

The second most predominant RdRp/capsid genotype in 2013 and from 2015 to 2017, GII.P12/GII.3 was also reported as the main NoV genotype in Chongqing (2011–2013), China [17]. Interestingly, this genotype was mainly observed in the Asia-Pacific region, implying that the GII.P12/GII.3 pandemic may have regional characteristics [17, 47, 48]. GII.P7/GII.6, as the second most prevalent genotype in 2014, was reported in a few cases elsewhere [46, 49]. In addition, many other rare RdRp/capsid strains, such as GII.Pe/GII.3 and GII.P16/GII.2, were detected in our study. Among these strains, the GII.P16/GII.2 strain was observed only in 2017; however, this strain has become the main genotype in Japan, France, Hong Kong, and Taiwan, as well as several other cities in China during 2016 and 2017 [17, 50–54]. Consequently, it is necessary to continuously monitor those rare strains in Shanghai.

In epidemiological investigations of children of different ages, the distribution of NoV RdRp/capsid genotypes varied in the different age groups. In our study, GII.Pe/GII.4-Sydney_2012 was detected in all age groups, while other genotypes were not. Some genotypes were only detected in one age group. These results may imply that the infection of some NoV RdRp/capsid genotypes was age specific. However, more and longer surveillance of the epidemiology of NoVs in children of different ages should be conducted to illustrate this phenomenon.

## Conclusions

In conclusion, our study has demonstrated the epidemiology and great genetic diversity of NoV GII combination genotypes in outpatient children less than 5 years old in Shanghai from 2012 to 2017. Many discordant RdRp/capsid genotypes were detected in our study and were reported for the first time in this area. Thus, continuous monitoring of NoV RdRp/capsid genotypes is needed to predict the emergence of new pandemic strains and guide the selection of norovirus vaccine strains in Shanghai.

## Data Availability

The datasets used in the current study are available from the corresponding author on reasonable request.

## Abbreviations

AGE: Acute gastroenteritis
NoVs: Noroviruses
ORFs: Open reading frames
RdRp: RNA-dependent RNA polymerase
